# Supplementary material for: Clinical Implications of the FLT3-ITD Allelic Ratio in Acute Myeloid Leukemia in the Context of an Allogeneic Stem Cell Transplantation
Source: Cancers (Basel). 2023 Feb 18;15(4):1312. doi: 10.3390/cancers15041312 (PMC9954047; doi:10.3390/cancers15041312)
Supplement: Supplementary file 1 [file cancers-15-01312-s001.zip › cancers-2156064-supplementary.pdf]

SUPPLEMENTARY INFORMATION

Clinical implications of the *FLT3*-ITD allelic ratio in acute myeloid leukemia in the context of an allogeneic stem cell transplantation, Jentzsch *et al*.

Induction therapy protocols of patients in the outcome set

In the subgroup of acute myeloid leukemia (AML) patients younger than 60 years at diagnosis (n=56), 41 patients received chemotherapy according to the AML 2002 study (OSHO #061),[1] three patients received 7+3 alone, five patients received chemotherapy within the Ratify trial (2 verum, 3 placebo),[2] one patient received 7+3 with Midostaurin, five patients were treated within the Quantum first trial (ClinicalTrials.gov Identifier: NCT02668653), and one patient was diagnosed with AML as a child and treated within the AML BFM-2014 study.[3]

Among AML patients older than 60 years at diagnosis (n=38), 25 patients were treated within the AML 2004 study (OSHO #069),[4] 9 patients were treated within the OSHO #083 protocol, two patients were treated within the Quantum first trial (ClinicalTrials.gov Identifier: NCT02668653), one patient received CPX-351[5] and one patient received 7+3 with Midostaurin.

Allogeneic HSCT in the outcome set

The majority of patients (n=47; 51%) received non-myeloablative (NMA) peripheral blood HSCT with 3x30 mg/m^2 Fludarabine and 2 Gy total body irradiation (TBI).[7] 30 patients (33%) received myeloablative conditioning (MAC) consisting of either 2x60 mg/kg body weight cyclophosphamide and 12 Gy TBI (n=27) or 5x30 mg/m^2 fludarabine and 8 Gy TBI (n=2). Seventeen patients (18%) received reduced intensity conditioning (RIC) consisting of either busulfan (8 mg/kg orally or 6.4 mg/kg intravenously) and 5x30 mg^2 fludarabine (n=2),[8] fludarabine and melphalan (n=2),[9] fludarabine, thiothepa and melphalan (n=2)[10] or FLAMSA-based conditioning (n=11).[11]

Definition of complete remission

CR was defined as the presence of <5% of blasts in bone marrow (BM), neutrophils >1.0 x 10^9/L, platelets >100 x 10^9/L, absence of blasts with Auer rods, independence of blood transfusion and no extramedullary disease.[14] CR with incomplete peripheral recovery (CRi) was defined as CR with platelets <100 x 10^9/L or neutrophils <1.0 x 10^9/L. In patients receiving allogeneic HSCT, the presence of CR or CRi was confirmed within 28 days prior to HSCT by bone marrow and peripheral blood analysis.

Multivariate analyses

Multivariate proportional hazard models were constructed for CIR, and OS to evaluate the impact of the *FLT3*-ITD allelic ratio in AML patients undergoing allogeneic HSCT by backward adjusting for other variables. The following variables were considered for multivariate analyses: sex, disease origin (*de novo vs* secondary), cytogenetic risk, *FLT3*-TKD and *NPM1* mutation status, age at HSCT, remission at HSCT (CR/CRi *vs* no CR/CRi), the MRD status at HSCT (MRD^{neg} *vs* MRD^{pos}), the number of remission at HSCT (first *vs* second), the HCT-CI risk score (0 *vs* 1/2 *vs* 3 or more points), cytomegalovirus (CMV) status of recipient and donor (high-risk [+/-] *vs* all others), donor type (matched related *vs* matched unrelated *vs* mismatched unrelated), and sex of the donor (female into male *vs* all others). Of these, variables significant at α=.10 in univariate analyses were considered for multivariable analyses. For all endpoints, hazard ratios with their corresponding 95% confidence intervals are indicated for every significant prognostic factor of the final model.

ddPCR Assays for hotspot *FLT3* D835 mutations

Primers and Probes were purchased from Biomers (Ulm, Germany); sequences are reported in Supplementary Table S1. Primer and probe design was performed using Primer 3 (https://primer3.ut.ee, version 4.1.0). The droplets were generated using the Automated Droplet Generator (BioRad).

For the *FLT3* D835 ddPCR following PCR conditions were used: initial denaturation (95° C for 10 minutes) followed by 40 cycles (denaturation 94° C for 30 seconds; annealing/extension 55° C for 1

minute) with a ramp rate set to 1° C/min and a final extension (98° C for 10 min), leading to a product of 82 bp for gDNA and 131 bp for cDNA.

Immunophenotype according to the *FLT3*-ITD AR at diagnosis

Patients with a high *FLT3*-ITD AR presented with a distinct immunophenotype at diagnosis, including a higher CD34+/CD38- cell burden (*P*<.001), a higher expression of antigens indicating myeloid differentiation (as CD13, *P*<.001; CD33, *P*<.001; and by trend CD64, *P*=.06, Supplementary Table S2) and a lower expression of antigens indicating erythroid, lymphoid or thrombocytic differentiation (Glycophorin A, *P*=.001; CD2, *P*=.001 and CD61, *P*=.003).

SUPPLEMENTARY REFERENCES

1 Büchner T, Schlenk RF, Schaich M, Doḧner K, Krahl R, Krauter J *et al.* Acute Myeloid Leukemia (AML): Different treatment strategies versus a common standard arm - Combined prospective analysis by the German AML Intergroup. *J Clin Oncol* 2012; **30**: 3604–3610.

2 Stone RM, Mandrekar SJ, Sanford BL, Laumann K, Geyer S, Bloomfield CD *et al.* Midostaurin plus chemotherapy for acute myeloid leukemia with a FLT3 Mutation. *N Engl J Med* 2017; **377**: 454–464.

3 Creutzig U, Zimmermann M, Bourquin JP, Dworzak MN, Fleischhack G, Graf N *et al.* Randomized trial comparing liposomal daunorubicin with idarubicin as induction for pediatric acute myeloid leukemia: Results from study AML-BFM 2004. *Blood* 2013; **122**: 37–43.

4 Niederwieser D, Hoffmann VS, Pfirrmann M, Al-Ali HK, Schwind S, Vucinic V *et al.* Comparison of Treatment Strategies in Patients over 60 Years with AML: Final Analysis of a Prospective Randomized German AML Intergroup Study. [abstract]. In: *Blood*. 2016, p 1066.

5 Lancet JE, Uy GL, Cortes JE, Newell LF, Lin TL, Ritchie EK *et al.* Cpx-351 (cytarabine and daunorubicin) liposome for injection versus conventional cytarabine plus daunorubicin in older patients with newly diagnosed secondary acute myeloid leukemia. *J Clin Oncol* 2018; **36**: 2684–2692.

6 Perl AE, Martinelli G, Cortes JE, Neubauer A, Berman E, Paolini S *et al.* Gilteritinib or chemotherapy for relapsed or refractory FLT3-mutated AML. *N Engl J Med* 2019; **381**: 1728–1740.

7 McSweeney PA, Niederwieser D, Shizuru JA, Sandmaier BM, Molina AJ, Maloney DG *et al.* Hematopoietic cell transplantation in older patients with hematologic malignancies: Replacing high-dose cytotoxic therapy with graft-versus-tumor effects. *Blood* 2001; **97**: 3390–3400.

8 Kröger N, Iacobelli S, Franke GN, Platzbecker U, Uddin R, Hübel K *et al.* Dose-reduced versus standard conditioning followed by allogeneic stem-cell transplantation for patients with myelodysplastic syndrome: A prospective randomized phase III study of the EBMT (RICMAC Trial). *J Clin Oncol* 2017; **35**: 2157–2164.

9 Baron F, Labopin M, Peniket A, Jindra P, Afanasyev B, Sanz MA *et al.* Reduced-intensity conditioning with fludarabine and busulfan versus fludarabine and melphalan for patients with acute myeloid leukemia: A report from the Acute Leukemia Working Party of the European Group for Blood and Marrow Transplantation. *Cancer* 2015; **121**: 1048–1055.

10 Duque-Afonso J, Ihorst G, Waterhouse M, Zeiser R, Wäsch R, Bertz H *et al.* Comparison of

reduced-toxicity conditioning protocols using fludarabine, melphalan combined with thiotepa or carmustine in allogeneic hematopoietic cell transplantation. *Bone Marrow Transplant* 2020; : 1–11.

11 Pfrepper C, Klink A, Behre G, Schenk T, Franke G-N, Jentzsch M *et al.* Risk factors for outcome in refractory acute myeloid leukemia patients treated with a combination of fludarabine, cytarabine, and amsacrine followed by a reduced-intensity conditioning and allogeneic stem cell transplantation. *J Cancer Res Clin Oncol* 2016; **142**: 317–324.

12 Niederwieser D, Maris M, Shizuru JA, Petersdorf E, Hegenbart U, Sandmaier BM *et al.* Low-dose total body irradiation (TBI) and fludarabine followed by hematopoietic cell transplantation (HCT) from HLA-matched or mismatched unrelated donors and postgrafting immunosuppression with cyclosporine and mycophenolate mofetil (MMF) can induce dura. *Blood* 2003; **101**: 1620–1629.

13 Glucksberg H, Storb R, Fefer A, Buckner CD, Neiman PE, Clift RA *et al.* Clinical manifestations of graft-versus-host disease in human recipients of marrow from HL-A-matched sibling donors. 1974; : 295–304.

14 Döhner H, Estey EH, Amadori S, Appelbaum FR, Büchner T, Burnett AK *et al.* Diagnosis and management of acute myeloid leukemia in adults: Recommendations from an international expert panel, on behalf of the European LeukemiaNet. *Blood* 2010; **115**: 453–474.

15 Gray RJ. A Class of K-Sample Tests for Comparing the Cumulative Incidence of a Competing Risk. *Ann Stat* 1988; **16**: 1141–1154.

SUPPLEMENTARY TABLES

Supplementary Table S1: Clinic, and genetic characteristics of analyzed patients according to the applied consolidation treatment (allogeneic HSCT or chemotherapy alone).

	**Patients consolidated by allogeneic HSCT n=94**	**Patients consolidated with chemotherapy n=24**
Age at diagnosis, years		
median	55.8	67.0
range	14.3-75.3	42.0-82.3
Sex, n (%)		
male	38 (40)	13 (54)
female	56 (60)	11 (46)
Disease origin, n (%)		
secondary	14 (15)	5 (21)
de novo	80 (85)	19 (79)
Hemoglobin, g/dL		
median	9.0	8.8
range	5.3-13.2	5.5-13.5
Platelet count, x 10^9/L		
median	63	64
range	7-256	7-313
WBC, x 10^9/L		
median	27.5	18.0
range	0.6-385	1.0-217.6
Blood blasts, %		
median	50	45
range	0-98	0-92
BM blasts, %		
median	75	75
range	4.6-95	30-95
BM CD34+/CD38- burden, %		
median	1	0.7
range	0-75	0-19
BM CD33 expression, %		
median	86	81
range	1-98	5-99
Normal karyotype, n (%)		
absent	31 (36)	5 (22)
present	56 (64)	18 (78)
ELN2017 genetic risk group, n (%)		
favorable	20 (22)	10 (50)
intermediate	45. (51)	6 (30)
adverse	24 (37)	4 (20)
FLT3-ITD allelic ratio, %		
median	0.44	0.36
range	0.01-14.1	0.01-29.5
NPM1, n (%)		
wild-type	51 (54)	9 (38)
mutated	43 (46)	15 (62)
CEBPA, n (%)		
wild-type	75 (91)	14 (88)
mutated	7 (9)	2 (13)
FLT3-TKD, n (%)		
wild-type	75 (84)	22 (92)
mutated	14 (16)	2 (8)

Abbreviations: BM, bone marrow; ELN, European LeukemiaNet, WBC, white blood cell count.

Supplementary Table S2: Additional clinic, and flow cytometry characteristics at diagnosis for all patients according to *FLT3*-ITD allelic ratio (*high vs* low, 0.5 cut), n=118

	All patients n=118	low *FLT3*-ITD AR n=67	high *FLT3*-ITD AR n=51	*P*
**Morphology**				
FAB type, n (%)				
M0	0 (0)	0 (0)	0 (0)	NA
M1	12 (11)	5 (8)	7 (14)	.37
M2	56 (51)	32 (53)	24 (48)	.70
M4	24 (22)	14 (23)	10 (20)	.82
M4eo	2 (2)	2 (3)	0 (0)	.50
M5	14 (13)	6 (10)	8 (16)	.40
M6	1 (1)	1 (2)	0 (0)	NA
M7	1 (1)	0 (0)	1 (2)	NA
**Immunophenotype**				
BM CD117 expression, %				.84
median	43	43	41	
range	0-95	0-93	0.5-95	
BM CD38 expression, %				.39
median	82	78	85	
range	0.5-98	4-98	0.5-97	
BM CD45 expression, %				<.001
median	97	94	98	
range	30-100	30-99	65-100	
BM CD11b expression, %				.27
median	16	15	22	
range	1-95	1-95	1-89	
BM CD13 expression, %				<.001
median	74	61	84	
range	10-97	10-91	15-97	
BM CD15 expression, %				.91
median	40	39	41	
range	1-93	1-91	1-93	
BM CD65 expression, %				.87
median	23	22	25	
range	0.5-91	0.5-91	0.5-88	
BM CD14 expression, %				.93
median	2	2	2	
range	0.5-56	0.5-50	0.5-56	
BM CD64 expression, %				.06
median	30	24	39	
range	0.5-98	0.5-88	0.5-98	
BM CD61 expression, %				.003
median	3	5	1	
range	0.5-43	0.5-40	0.5-43	
BM Glycophorin A expression, %				.001
median	5	8	4	
range	0.5-50	0-50	0.5-36	
BM CD2 expression, %				.001
median	11	14	6	
range	1.5-67	2-97	1.5-67	
BM CD7 expression, %				.30

median	13	14	9	
range	1-94	2-94	1-93	
BM CD56 expression, %				.92
median	4	5	4	
range	0-96	0.5-63	1-96	

Abbreviations: *FAB, french american british; CD, cluster of differentiation.*

<u>Supplementary Table S3:</u> HSCT-associated characteristics for patients in the outcome set according to *FLT3*-ITD allelic ratio (*high vs* low, 0.5 cut), n=94

	All patients n=94	low *FLT3*-ITD AR n=53	high *FLT3*-ITD AR n=41	*P*
**Transplant-related characteristics**				
Conditioning regimen, n (%)				.89
MAC	32 (34)	17 (32)	15 (37)	
RIC	15 (16)	9 (17)	6 (15)	
NMA	47 (50)	27 (51)	20 (49)	
Remission status at HSCT, n (%)				.38
No CR/CRi	11 (12)	7 (13)	4 (10)	
CR/CRi1	62 (66)	37 (70)	25 (61)	
CRi/CRi2	21 (22)	9 (17)	12 (29)	
Use of FLT3 inhibitor, n (%)				.08
none	82 (87)	43 (81)	39 (95)	
yes	5 (5)	5 (9)	0 (0)	
unknown (blinded)	7 (7)	5 (9)	2 (5)	
donor type, n (%)				.71
HLA matched related	19 (20)	9 (17)	10 (24)	
HLA matched unrelated	54 (57)	32 (60)	22 (54)	
HLA mismatched	21 (22)	12 (23)	9 (22)	
donor & recipient sex, n (%)				.74
no female into male	83 (89)	47 (89)	36 (88)	
female into male	10 (11)	5 (11)	5 (12)	
CMV status, n (%)				.82
recipient + / donor –	68 (72)	39 (74)	29 (71)	
all others	26 (28)	14 (26)	12 (29)	
aGvHD ≥ grade 2, n (%)				.82
absent	55 (68)	30 (67)	25 (69)	
present	26 (32)	15 (33)	11 (31)	
cGvHD, n (%)				.63
absent	30 (48)	16 (44)	14 (52)	
limited	12 (19)	6 (17)	6 (22)	
extended	21 (33)	14 (39)	7 (26)	
**MRD status prior to HSCT**				
Pre-HSCT MRD, n (%)				.02
negative	22 (63)	13 (87)	9 (45)	
positive	13 (37)	2 (13)	11 (55)	

<u>Abbreviations:</u> aGvHD, acute graft versus host disease; AR, allelic ratio; cGvHD, chronic graft versus host disease; CMV, cytomegalovirus; CR, complete remission; CRi, CR with incomplete peripheral recovery; HLA, human leukocyte antigen; HCT-CI, hematopoietic cell transplantation comorbidity index; HSCT, hematopoietic stem cell transplantation; MRD, measurable residual disease.

<u>Supplementary Table S4:</u> *FLT3*-TKD primer/probe design for MRD assays

FLT3 **D835**	
Primer Forward gDNA	CACGGGAAAGTGGTGAAGAT
Primer Reverse gDNA	CATTGCCCCTGACAACATAG
Primer Forward cDNA	CACGGGAAAGTGGTGAAGAT
Primer Reverse cDNA	ATGCCTTCAAACAGGCTTTC
WT Probe (FAM labeled)	GGCT[+C][+G][+A][+G]ATATCATGAGT
p.D835Y c.2503G>T Probe (HEX labeled)	GGCT[+C][+G][+A][+T]ATATCATGAGT
p.D835H c.2503G>C Probe (HEX labeled)	GGCT[+C][+G][+A][+C]ATATCATGAGT
p.D835V c.2504A>T Probe (HEX labeled)	GGCT[+C][+G][+A]G[+T]TATCATGAGT
p.D835N c.2503G>A Probe (HEX labeled)	GGCT[+C][+G][+A][+A]ATATCATGAGT

<u>Supplementary Table S5:</u> Multivariate analyses

	Cumulative incidence of relapse/progression		Overall survival	
	**HR* (95% CI)**	***P***	**HR* (95% CI)**	***P***
**Patient sex (female *vs* male)**	0.23 (0.06-0.84)	.03	5.88 (1.55-22.28)	.009
**pre-HSCT remission status (MRD^{pos} *vs* MRD^{neg})**	13.2 (3.05-57.0)	<.001	-	-

<u>Abbreviations:</u> CI, confidence interval; HSCT, hematopoietic stem cell transplantation; MRD, measurable residual disease.

*HR, hazard ratio, <1 (>1) indicate lower (higher) risk of relapse for the first category listed for the dichotomous variables for the lower (higher) values of the continuous variables.

Variables considered in the models were those significant at α=0.10 in univariate analyses.

For CIR, these variables were patient sex, the MRD status at HSCT, and conditioning intensity (NMA/RIC *vs* MAC). For OS, these variables were patient sex, *FLT3*-TKD mutation status, morphologic remission at HSCT and the MRD status at HSCT.

Supplementary Figures

Supplementary Figure S1

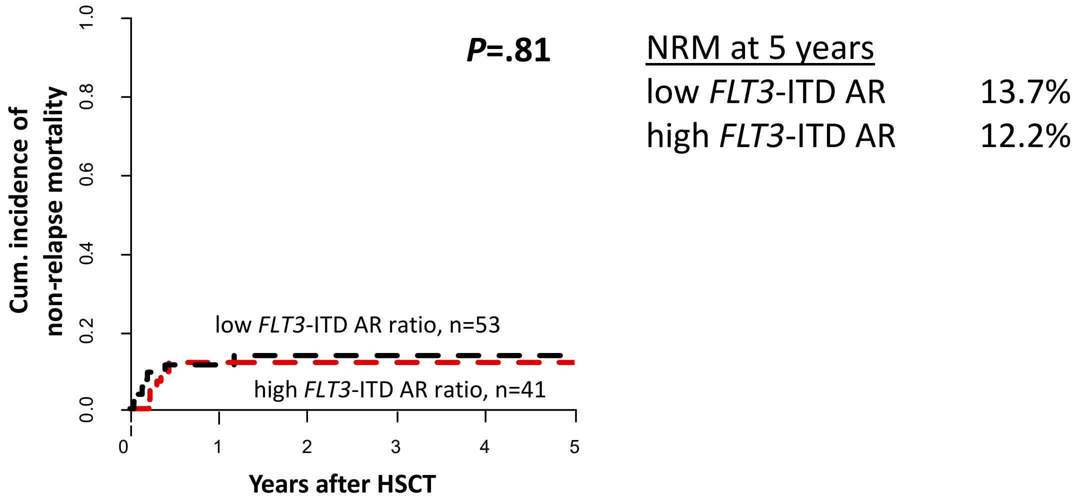

Supplementary Figure S1: Non-relapse mortality according to *FLT3*-ITD allelic ratio (high *vs*. low, 0.5 cut, n=94).

Supplementary Figure S2

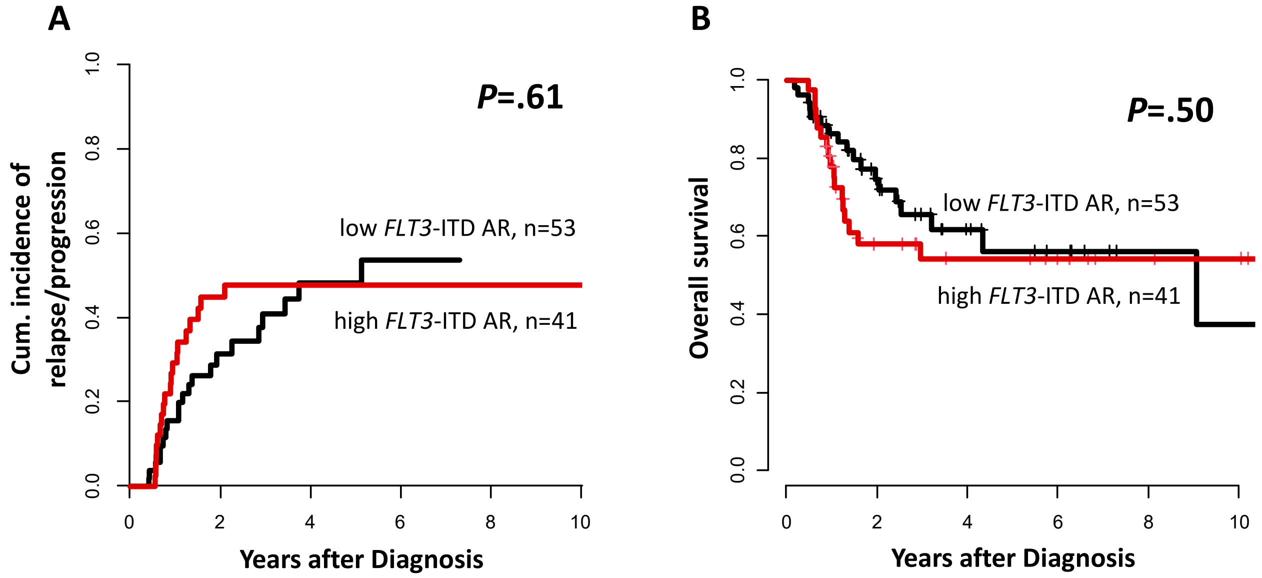

Supplementary Figure S2. Outcome according to *FLT3*-ITD allelic ratio (<0.5 *vs* ≥ 0.75) in patients receiving allogeneic HSCT, calculated from the time of diagnosis (n=94), (A) Cumulative incidence of relapse/progression and **(B)** Overall survival.

Supplementary Figure S3

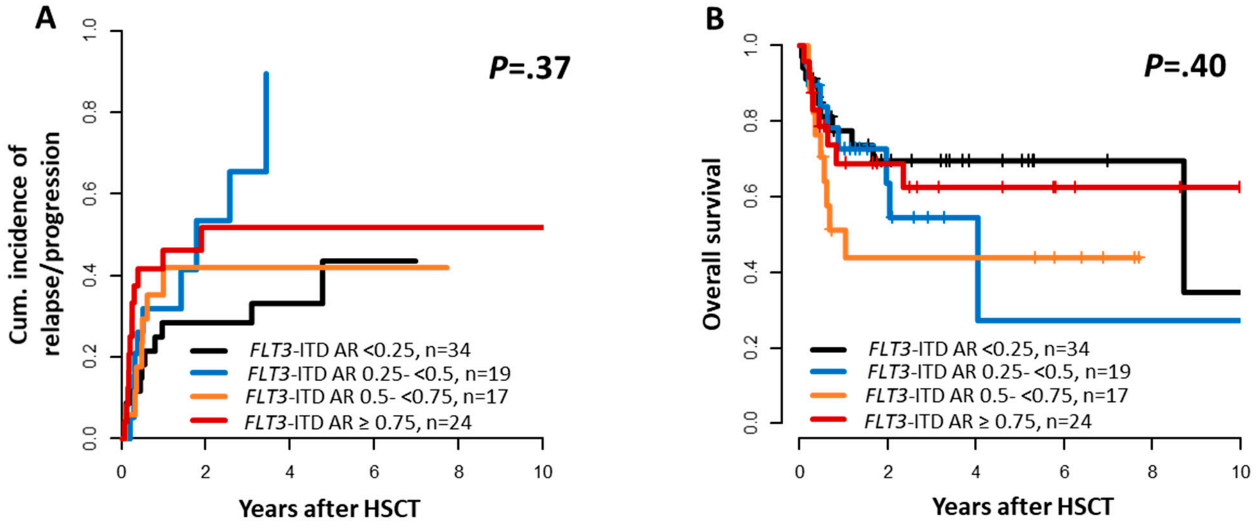

Supplementary Figure S3. Outcome according to *FLT3*-ITD allelic ratio (AR, <0.25 *vs* 0.25- <0.5 *vs* 0.5-0.75 *vs* ≥ 0.75) in patients receiving allogeneic HSCT (n=94), (A) Cumulative incidence of relapse/progression and **(B)** Overall survival.

Supplementary Figure S4

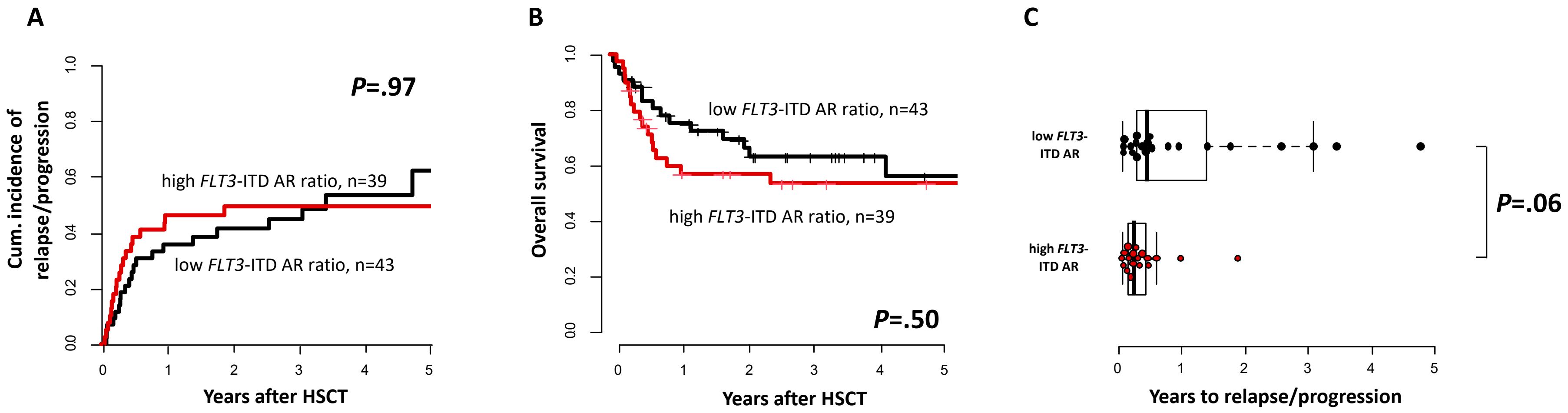

Supplementary Figure S4. Outcome according to *FLT3*-ITD allelic ratio (AR, high *vs*. low, 0.5 cut) in patients not treated with a FLT3 inhibitor prior to allogeneic HSCT (n=82), (A) Cumulative incidence of relapse/progression, **(B)** Overall survival and **(C)** Time to relapse in relapsing patients.

Supplementary Figure S5

Outcome according to *NPM1* mutation

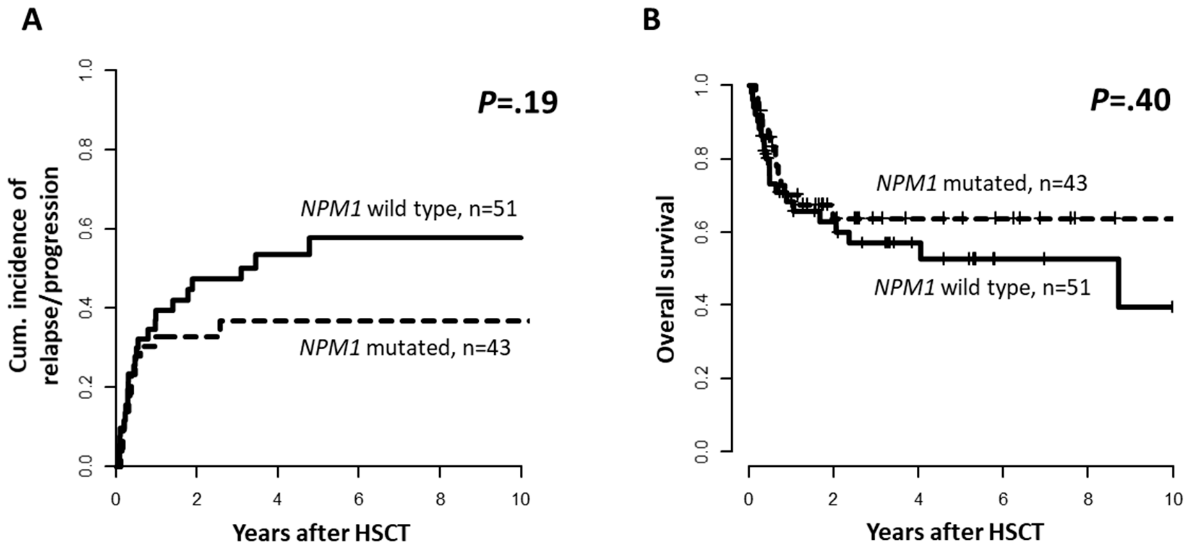

Outcome according to *NPM1* mutation and *FLT3*-ITD AR

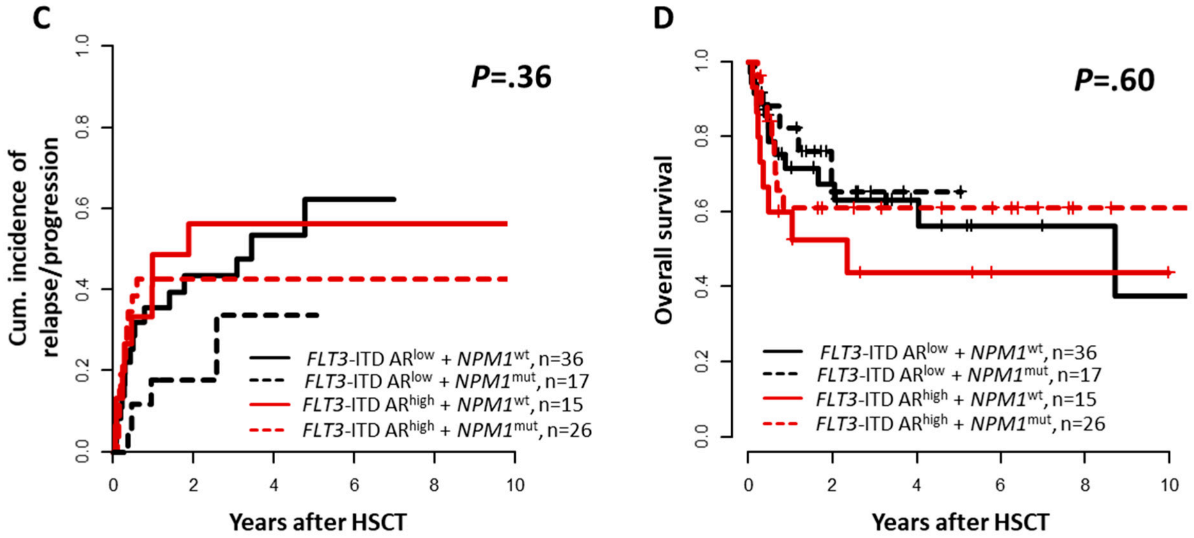

Supplementary Figure S5. Outcomes of patients harboring a *FLT3*-ITD undergoing allogeneic HSCT according to the mutational status of *NPM1* (A) Cumulative incidence of relapse/progression and **(B)** Overall survival**, as well as according to the mutational status of *NPM1* and the *FLT3*-ITD allelic ratio (AR, high *vs.* low, 0.5 cut) (C)** Cumulative incidence of relapse/progression and **(D)** Overall survival.

Supplementary Figure S6

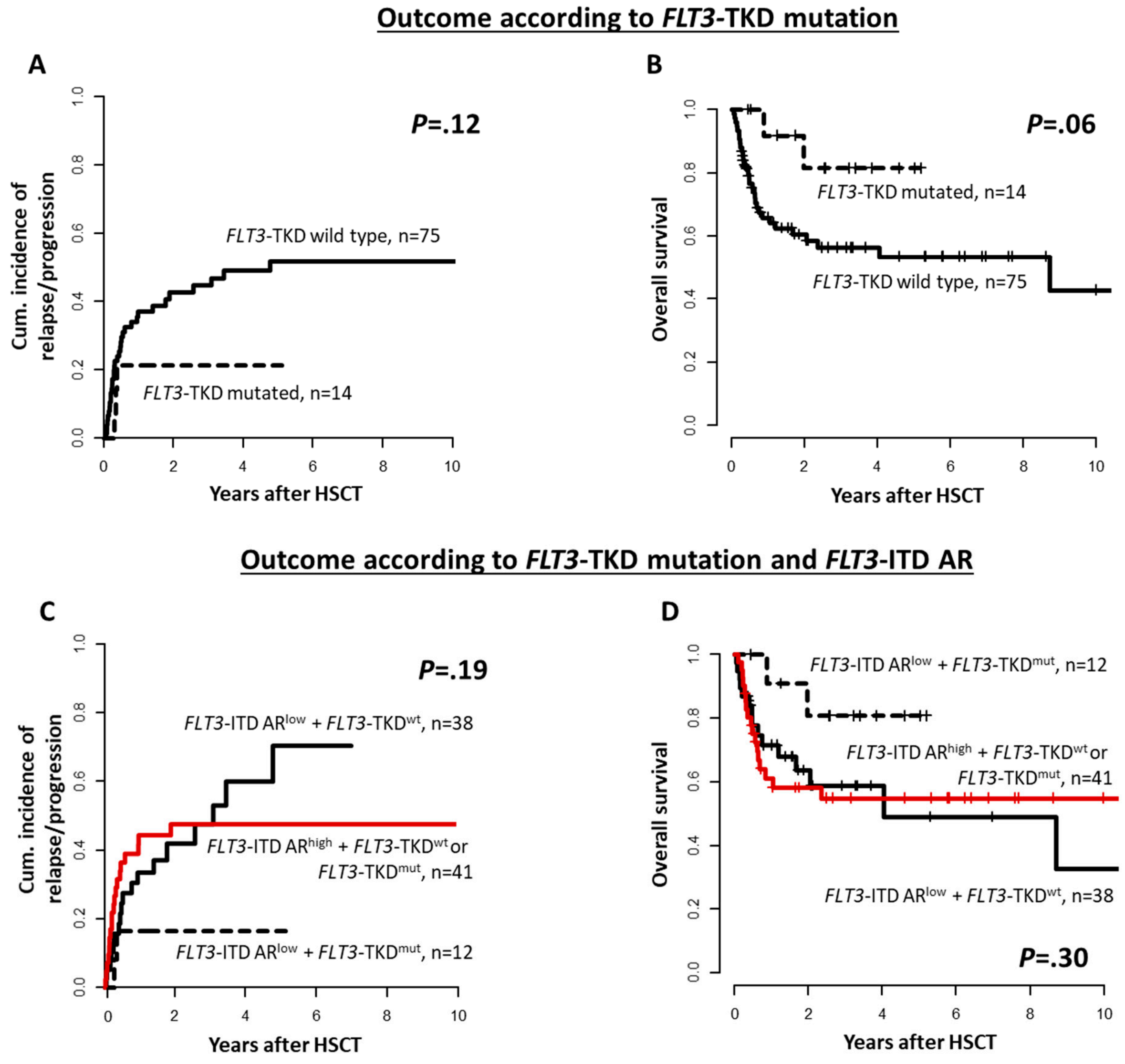

Supplementary Figure S6. Outcomes of patients harboring a *FLT3*-ITD undergoing allogeneic HSCT according to the mutational status of *FLT3*-TKD (A) Cumulative incidence of relapse/progression and **(B)** Overall survival**, as well as according to the mutational status of *FLT3*-TKD and the *FLT3*-ITD allelic ratio (AR, high *vs.* low, 0.5 cut) (C)** Cumulative incidence of relapse/progression and **(D)** Overall survival.

Supplementary Figure S7

Outcome according to the *FLT3*-ITD allelic ratio in patients after RIC- or NMA-HSCT

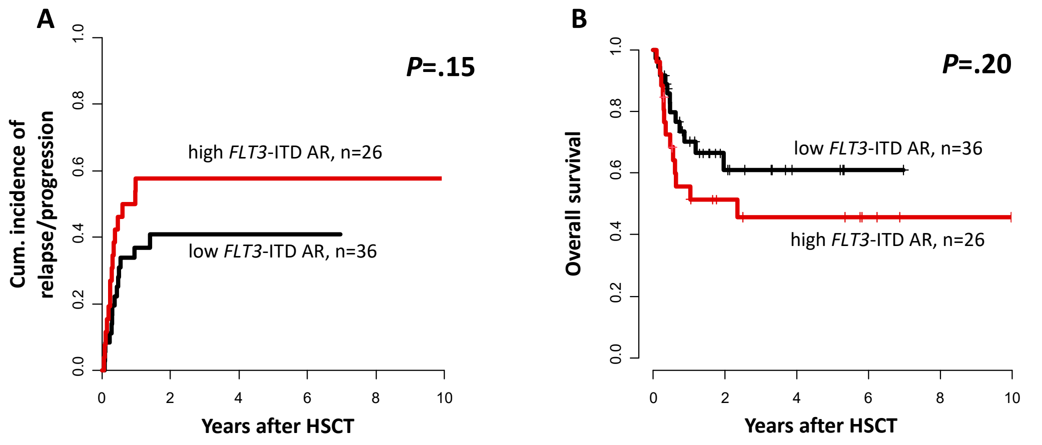

Outcome according to the *FLT3*-ITD allelic ratio in patients after MAC-HSCT

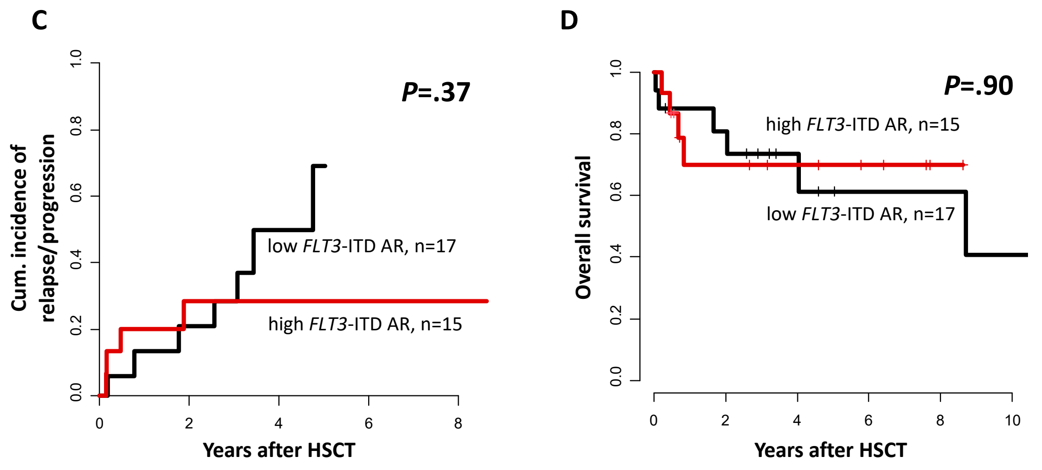

Supplementary Figure S7. Outcomes of patients harboring a *FLT3*-ITD undergoing allogeneic HSCT according to the *FLT3*-ITD allelic ratio (AR, high *vs.* low, 0.5 cut) and the conditioning regimen used. **(A)** Cumulative incidence of relapse/progression and **(B)** Overall survival in patients receiving non-myeloablative or reduced-intensity conditioning, and **(C)** Cumulative incidence of relapse/progression and **(D)** Overall survival in patients receiving myeloablative conditioning.
